# The Diagnosis of Cutaneous Cancers

**Published:** 1903-04

**Authors:** F. H. Beadles

**Affiliations:** Richmond, Va.; Lecturer on Diseases of the Skin and Professor of Pharmacognosy and Botany, Medical College of Virginia


### THE DIAGNOSIS OF CUTANEOUS CANCERS.*

BY F. H. BEADLES, M.D., RICHMOND, VA.

Lecturer on Diseases of the Skin and Professor of Pharmacognosy and Botany, Medical College of Virginia.

There can be no question that the establishment of an accurate diagnosis is essential to the successful management of morbd growths of the skin. It is a common error that the sole requisite for determining such a diagnosis is the exhibition of the affected portion of the integument, and that the physician shall be able, by merely viewing the surface for a few minutes, to pronounce definitely the nature of the disease and determine the therapeutic measures to be adopted. But much more than this is essential, as much so as in the investigation of diseases involving any other organ of the body.

Three almost indispensable aids in obtaining a correct diagnosis are: a good light, a good eye and a microscope.

If possible, secure a history, past and present, of the physical condition of the patient, his parents and his children. Note his age and occupation, and whether married or single. Unless these facts are taken into consideration, the management of the case must be haphazard, unscientific and culpable.

The principal diagnostic points of cutaneous cancer are: 1. The advanced age of the patient. 2. The beginning of the disease as a wart, mole, nodular or scaly patch. 3. The usual slow progress of the disease. 4. The single character of the growth. 5. The striking appearance of its border, which is of a pearly hue with roll-like elevations or a hard elevated infiltration. 6. The scant, and in the later stages viscid, discharge, frequently streaked with blood. 7. The situation of the affected portion, usually the nose, eyelids and frequently involving the other portions of the face.

The conditions which simulate cutaneous cancer are: Syphilis, lupus and Baelzer's disease.

*Read before the Richmond Academy of Medicine and Surgery, February 24, 1903.

The syphiloderm, which bears a most striking likeness to a skin cancer, is the tubercular ulcerating form. Its distinguishing features are: 1. Its more rapid growth. 2. Its multiple lesions consisting of several superficial ulcerations, rarely round in shape but segmented or irregularly circinate. 3. The presence of tubercles which have not undergone destructive changes. 4. Its usually free discharge of a distinctly pustular character. A pronounced symptom of a syphilitic ulceration is a strong tendency to reparative cicatrization, due partly to the exhaustion of the infective poison and partly to an insufficient but modifying treatment.

Lupus vulgaris is a disease which usually develops in youth and rarely after the age of thirty-five. It presents a tubercle of a reddish-brown color and of a soft consistency. In the earlier period of its career, it is more diffuse than cancer, while in the ulcerating stage its edges are often bordered by non-ulcerating papules. The presence of the tubercle bacillus is revealed by the use of the microscope.

Baelzer's disease was, for a long time, rather confusing to me, as the literature on the subject is rather limited. It is a chronic affection of the mucous glands of the lip, marked by an indolent swelling and infiltration of the periglandular tissue, and a slow ulcerating process extending from above downward. There is also present a catarrhal inflammation of the mucous membrane of the lip. The distinctive points of the disease are: 1. The absence of general systemic disturbance. 2. The absence of implication of the neighboring lymphatic glands. 3. Its ready response to simple treatment, as the application of tincture of iodine.

